# Risk-Stratified Treatment in Chronic Lymphocytic Leukemia

**Published:** 2016-04-01

**Authors:** Deborah M. Stephens, Amy L. Goodrich

**Affiliations:** Huntsman Cancer Institute, Salt Lake City, Utah, and Johns Hopkins University, Baltimore, Maryland

Novel agents for chronic lymphocytic leukemia (CLL) are extending survival for these patients, calling for clinicians to understand the individual patient’s risk and prognosis and his or her long-term needs and optimal management, according to Deborah M. Stephens, DO, of Huntsman Cancer Institute, Salt Lake City, Utah, and Amy L. Goodrich, MSN, CRNP, of Johns Hopkins University, Baltimore, Maryland.

At JADPRO Live at APSHO, Dr. Stephens commented, "This is an exciting time for patients with CLL. We have many novel therapies and more to come, and they are keeping our patients alive."

## PATIENT WORK-UP

Ms. Goodrich described the essential components of the work-up of suspected CLL: (1) laboratory evaluations, including complete blood count (CBC) with differential, peripheral blood smear, comprehensive panel, (2) history, including performance status and presence of B symptoms, (3) physical examination, including nodal regions, Waldeyer’s ring and hepato/splenomegaly, and (4) definitive pathology, including peripheral blood flow cytometry; if flow cytometry is not diagnostic, lymph node biopsy can be considered. Additional work-up is warranted under certain conditions.

Dr. Stephens emphasized that a bone marrow biopsy is not needed to confirm a diagnosis if peripheral blood flow cytometry is indicative of CLL. "The unique thing about this cancer is that staging is based on clinical exam and laboratory findings," she said.

## PROGNOSTIC FACTORS

Determination of prognosis is important to patients and helps physicians select treatment. Clinicians should focus on tests "that will be most prognostic and will tell the patient what the next few years of their lives will be like," Dr. Stephens suggested.

"The first and easiest thing to determine is clinical stage," she said. Stage is strongly associated with survival. Mortality risk essentially doubles for stage I/II vs. stage 0, and for stage III/IV vs. I/II (Pflug et al., 2014; see Table).

**Table:**
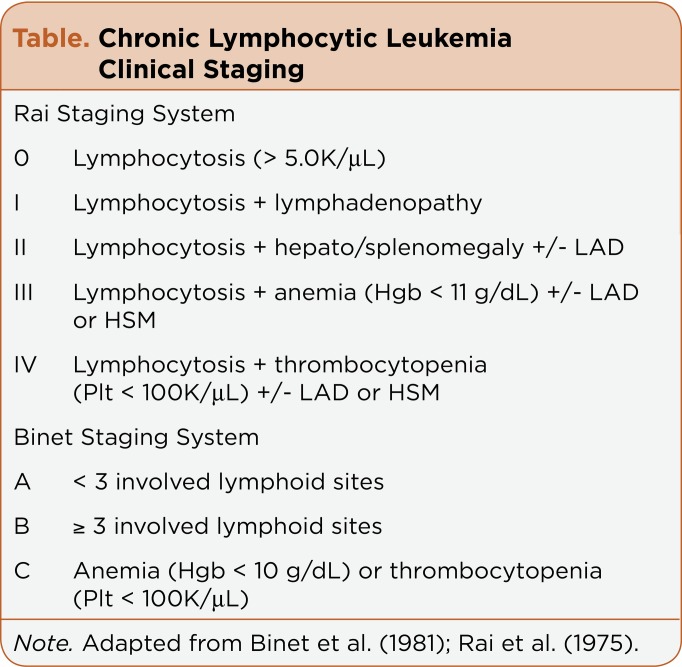
Chronic Lymphocytic Leukemia Clinical Staging

"Stage 0 patients have extended survival, up to 12 years," she noted. "Stage III/IV at diagnosis still have a good survival, but it’s limited to about 8 years."

The mutation status of immunoglobulin variable heavy chain (IgVH) is important in prognosis. Unmutated IgVH heralds aggressive disease. While patients with mutated IgVH have an expected survival of about 25 years and 80% may never need treatment, average patients with unmutated IgVH live about 9 years, and virtually all these patients will need treatment.

"This prognostic factor is constant over time. At diagnosis, it can give the patient information they can use for planning," Dr. Stephens said.

Fluorescence situ hybridization (FISH) is used to probe for the most common and significant tumor mutations in CLL, including deletions 17p, 13q, and 11q, and trisomy 12. Karyotype, which examines chromosomes, delves further and detects additional abnormalities in up to one-third of patients.

"Complex karyotype," indicated by more than 3 chromosomal abnormalities, is strongly associated with poor treatment response and poor prognosis, she noted.

Most important for prognosis is del(17p), which heralds the most aggressive disease. "The presence or absence of deletion 17p helps me choose treatment," Dr. Stephens said.

Since mutational status and clonal evolution change over time and also with treatment (usually toward higher-risk mutations), karyotype and FISH should be repeated over the course of the disease and treatment tailored accordingly.

## TREATMENT OF CLL

Treatment should be considered when patients develop significant disease-related symptoms, progressive bulky disease, threatened end-organ dysfunction, or progressive anemia or thrombocytopenia. In select patients with stable and mild cytopenias (hemoglobin < 11 g/dL, platelets < 100,000/µL), continued observation may be appropriate, according to Ms. Goodrich.

More important than absolute platelet count is "the pace of change," she added.

## INITIATING TREATMENT

When determining initial treatment, clinicians should first evaluate the patient’s risk based on lab values and clinical features, confirm the presence or absence of del(17p), and evaluate performance status. Treatment is designed based on these key factors.

For patients lacking del(17p) who cannot tolerate aggressive therapy, three anti-CD21 monoclonal antibodies are available. Regimens include obinutuzimab (Gazyva) with or without chlorambucil (Leukeran), ofatumumab (Arzerra) plus chlorambucil, rituximab (Rituxan) plus chlorambucil, chlorambucil alone, pulse steroids and, for some patients, bendamustine plus rituximab (BR).

In a recent study, obinutuzumab plus chlorambucil significantly improved median progression-free survival over rituximab/chlorambucil (26.7 vs. 16.3 months; Goede et al., 2014; see Figure).

**Figure:**
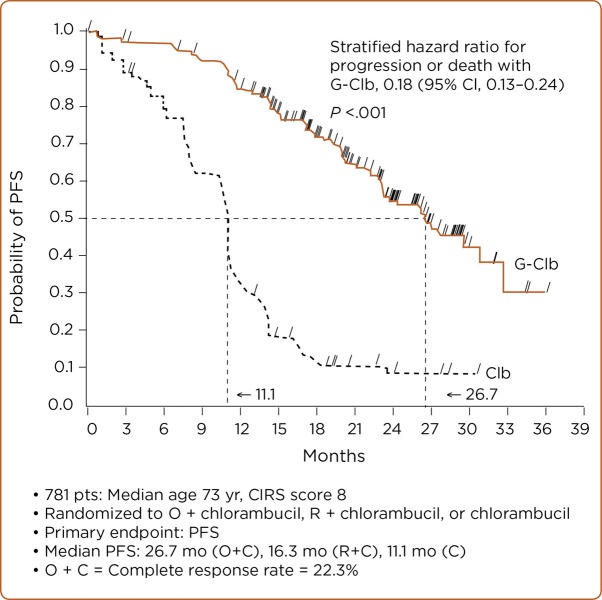
Obinutuzumab plus chlorambucil. Adapted from Goede et al. (2014).

For patients who can tolerate more aggressive treatment, the standard regimens are fludarabine/rituximab (FR), FR plus cyclophosphamide (FCR), pentostatin/rituximab/cyclophosphamide (PCR) and BR. Emerging data suggest that in younger patients FCR improves progression-free survival compared to BR, she indicated.

For relapsed disease, patients are still classified according to del(17p) mutation status and fitness for aggressive treatment. Relapsed patients with del(17p) who cannot tolerate aggressive therapy have several good options, including lenalidomide (Revlimid) with or without rituximab, and the newer B-cell signaling inhibitors, which are ibrutinib (Imbruvica) alone or idelalisib (Zydelig) with or without rituximab.

"We are very fortunate today to have these less toxic therapies," Dr. Stephens said.

Patients who can tolerate aggressive treatment could also receive high-dose methylprednisolone with or without rituximab, alemtuzumab with or without rituximab, or the potent regimen of oxaliplatin/fludarabine/cytarabine/rituximab.

BR is not considered a good treatment for patients with relapsed del(17p), based on a study demonstrating very poor response rates, event-free survival, and overall survival with this regimen in that subset (Fischer et al., 2011).

## B-CELL RECEPTOR SIGNALING INHIBITORS

The oral B-cell receptor signaling inhibitors—ibrutinib and idelalisib—are "very exciting advances in patients with CLL," Dr. Stephens commented.

In key clinical trials, ibrutinib led to a 30-month overall survival rate of 79%, and 65% among the del(17p) subset (Byrd et al., 2013). Idelalisib in combination with rituximab led to an 81% response rate and a median progression-free survival that was not reached, compared to 5.5 months with rituximab alone (Furman et al., 2014).

Clinicians are becoming familiar with the transient lymphocytosis that emerges soon after treatment initiation and peaks in about 2 months. This is thought to be due to redistribution of CLL lymphocytes from the lymph nodes into the peripheral circulation.

"Lymphocytosis is not an adverse event and is not an indication of disease progression," Dr. Stephens emphasized. "The drugs should not be stopped if patients are responding."

## MONITORING FOR ADVERSE EVENTS

"Clinicians should know the exclusion criteria and potential adverse effects of the novel therapeutic agents," Dr. Stephens said.

Common adverse events with ibrutinib are cytopenias, diarrhea, fatigue, musculoskeletal pain, rash, nausea, and fever. For a grade ≥ 3 event, she had the following recommendations:

- Temporarily discontinue ibrutinib. Resume when the side effect resolves to
- ≤ grade 1 and resume at the original dose.
- For second and third occurrences, use clinical judgment; the dose can be reduced by 140 mg (1 tablet) per occurrence.
- For a fourth occurrence, discontinue ibrutinib.

Clinicians should be aware of drug interactions with CYP3A inhibitors and inducers, and of hemorrhage risk. Ibrutinib should not be given to patients taking warfarin (substitute idelalisib); it should be held for a few days before and after tooth extraction and planned surgical procedures. Clinical judgment is called for in managing patients with spontaneous bleeds while on ibrutinib, Dr. Stephens said.

With idelalisib plus rituximab, common side effects are cytopenias, transaminitis, pneumonia, diarrhea, nausea, and rash.

Diarrhea can be inflammatory and in 14% of cases "serious to fatal." Dietary modifications and loperamide may be enough for mild cases; for severe cases, idelalisib should be held and resumed at 100 mg bid. Transaminitis can be severe and may warrant discontinuation of treatment if holding the dose and resuming at a lower dose is not effective. For cytopenias, clinicians should monitor counts and lower idelalisib dose if necessary.

For all patients with CLL, Immunoglobulin G (IgG) deficiency can occur, with its risk increasing over time. This potentiates infection, which is a leading cause of death in CLL patients.

"Looking for IgG deficiency and replacing IgG is worth the time and effort," Ms. Goodrich added.

## ROLE OF THE ADVANCED PRACTITIONER

Advanced practitioners can be the lynchpin of the multidisciplinary management that CLL patients need, the speakers said.

"These patients have multiple health problems, and psychosocial and financial needs as well, which you need to find out about," Ms. Goodrich said. "CLL patients often get caught in the gaps in our healthcare system. It’s the advanced practice clinician who keeps these patients duct-taped together."

Frequent monitoring for and tight management of side effects, along with patient education, greatly helps patients adhere to these new oral agents, Ms. Goodrich emphasized.
